# Retinal Spectral Domain Optical Coherence Tomography in Early Atrophic Age-Related Macular Degeneration (AMD) and a New Metric for Objective Evaluation of the Efficacy of Ocular Nutrition

**DOI:** 10.3390/nu4121812

**Published:** 2012-11-27

**Authors:** Stuart Richer, Jane Cho, William Stiles, Marc Levin, James S. Wrobel, Michael Sinai, Carla Thomas

**Affiliations:** 1 Eye Clinic, James A Lovell Federal Health Care Center, North Chicago, IL 60064, USA; Email: jane.cho@my.rfums.org (J.C.); ilovemabel@aol.com (W.S.); medpat@comcast.net (M.L.); carla.thomas2@va.gov (C.T.); 2 Family & Preventive Medicine, RFUMS Chicago Medical School, North Chicago, IL 60064, USA; 3 Internal Medicine, Podiatry Services, University of Michigan, Ann Arbor, MI 48105, USA; Email: jswrobel@med.umich.edu; 4 Optovue Inc., Fremont, CA 94538, USA; Email: mike_sinai@optovue.com

**Keywords:** atrophic age-related macular degeneration (AMD), spectral domain OCT (SD OCT), contrast sensitivity function (CSF), extrafoveal blue-cone increment thresholds

## Abstract

Purpose: A challenge in ocular preventive medicine is identification of patients with early pathological retinal damage that might benefit from nutritional intervention. The purpose of this study is to evaluate retinal thinning (RT) in early atrophic age-related macular degeneration (AMD) against visual function data from the Zeaxanthin and Visual Function (ZVF) randomized double masked placebo controlled clinical trial (FDA IND #78973). Methods: Retrospective, observational case series of medical center veterans with minimal visible AMD retinopathy (AREDS Report #18 simplified grading 1.4/4.0 bilateral retinopathy). Foveal and extra-foveal four quadrant SDOCT RT measurements were evaluated in *n* = 54 clinical and ZVF AMD patients. RT by age was determined and compared to the OptoVue SD OCT normative database. RT by quadrant in a subset of *n* = 29 ZVF patients was correlated with contrast sensitivity and parafoveal blue cone increment thresholds. Results: Foveal RT in AMD patients and non-AMD patients was preserved with age. Extrafoveal regions, however, showed significant slope differences between AMD patients and non-AMD patients, with the superior and nasal quadrants most vulnerable to retinal thinning (sup quad: −5.5 μm/decade thinning *vs.* Non-AMD: −1.1 μm/decade, *P* < 0.02; nasal quad: −5.0 μm/decade thinning *vs.* Non-AMD: −1.0 μm/decade, *P* < 0.04). Two measures of extrafoveal visual deterioration were correlated: A significant inverse correlation between % RT and contrast sensitivity (*r* = −0.33, *P* = 0.01, 2 Tailed Paired T) and an elevated extrafoveal increment blue cone threshold (*r* = +0.34, *P* = 0.01, 2 Tailed T). Additional SD OCT RT data for the non-AMD oldest age group (ages 82–91) is needed to fully substantiate the model. Conclusion: A simple new SD OCT clinical metric called “% extra-foveal RT” correlates well with functional visual loss in early AMD patients having minimal visible retinopathy. This metric can be used to follow the effect of repleting ocular nutrients, such as zinc, antioxidants, carotenoids, *n*-3 essential fats , resveratrol and vitamin D.

## 1. Introduction

Age-related macular degeneration (AMD) is the leading cause of vision loss in both developed and developing countries [1,2,3]. The initial clinical stereoscopic ophthalmoscopic manifestations of the dry form of the disease are drusen formation and RPE hypo/hyper pigmentation. These signs have been used in large scale “ocular nutrient” studies such as AREDS I and II to monitor disease and stratify risk of progression. Yet much pathology is hidden below the surface of the retina. Classic fundus observation and photography even with stereoscopic enhancement by a highly trained physician often reveals merely the gross manifestations of moderate and advanced disease. Emerging sensitive, new technologies include retinal pigment epithelium autofluorescence imaging [4], multi spectral retinal imaging (*i.e*., Annidis Health Systems, Ottawa, Canada) [5], and high resolution spectral domain OCT imaging for more subtle changes of the retina and choroid [6]. Beyond imaging, lipofuscin is the most consistent and phylogenically constant marker of cellular aging that can be useful for managing AMD [7]. Furthermore, autofluorescence of the A2E fluorophore within the RPE is utilized within AREDS 2 to evaluate geographic atrophic edge expansion [8]. These new technologies, when broadly evaluated in conjunction with preventive medicine/nutritional intervention, may assist eye physicians in preventing the development of moderate and advanced catastrophic AMD.

The Optovue^®^ RTVue (Fremont, CA) was the first commercially available spectral/Fourier domain optical coherence tomography (SD OCT) instrument in the United States. This SD OCT utilizes a low-coherence super luminescent diode light source at a near infrared wavelength of 840 nm (bandwidth 50 nm), and is capable of achieving 5 µm axial tissue resolution. The MM5 scan pattern provides full retinal thickness measurements in the macula as well as nine summary parameters from a 9-zone ETDRS grid. 

**Figure 1:**
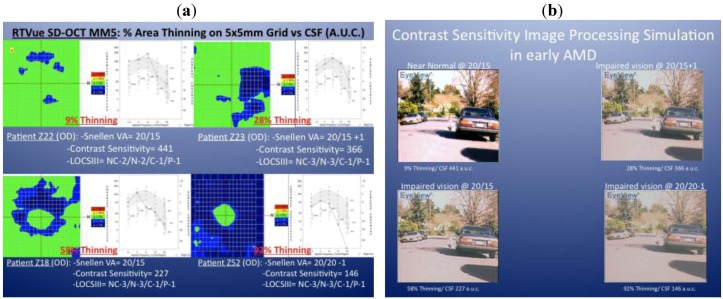
Retinal thinning, declining contrast sensitivity and simulated impaired driver visibility. (**a**) Spectral Domain OCT MM5 plots of early atrophic AMD phakic retinas from four eyes of four different patients (subject Z229% extra-foveal thinning (EFT); Z23 28% EFT; Z18 58% EFT and Z52 92% EFT) in the Zeaxanthin and Vision Function Study (ZVF) FDA IND #78973—see [10]. These subjects all had similar lens opacification by LOCS III analysis and 20/20 or better ETDRS visual acuity. Adjacent CSF plots indicate declining contrast sensitivity with increasing loss of parafoveal retinal thickness. (**b**) Image simulation of a traffic scene of a little girl next to a car computed from each CSF function shown to the left. Images correlate with the monocular CSF of these four patients (clockwise upper left: Z22, Z23, Z18 and Z52), using image processing software (Functional Vision Analyzer^®^) donated by Stereo Optical, Inc., Chicago, IL.

Clinical observations stimulated the design of this study. We observed that patients with very early, and often subclinical atrophic AMD, display various degrees of parafoveal thinning using the SD OCT MM5 protocol. For example, Figure 1a depicts the right eye MM5 and contrast sensitivity function (CSF) of four patients with early atrophic AMD, mild and similar lens opacification rating using the LOCS III grading system [9] and 20/20 or better Snellen acuity. The green areas on the thickness maps show regions that have within normal thickness values (compared to the RTVue normative database). The blue areas show regions that have significant thinning (*P* < 0.05). Note that with increasing parafoveal geographic thinning, there is a dramatic declining contrast sensitivity function (CSF) “Area under the curve” (AUC) value. None of these four representative eyes had additional pathology that would decrease the CSF function, *i.e.*, uncorrected refractive error, clinically significant cataract, diabetes, glaucoma or neurodegenerative disease. Figure 1b depicts an image simulation of a high-risk driver-pedestrian traffic scene generated when each of these four CSF functions is analyzed with image processing software (Functional Vision Analyzer^®^, Stereo Optical, Inc., Chicago, IL). The last driver in particular, with 92% parafoveal + perifoveal thinning preserves his central foveal thickness, has 20/20 Snellen acuity yet profound CSF AUC loss, profound distance visual disability and profound simulated road-scene image degradation. This clinical observation led us to the hypothesis that retinal thinning is quickly measured by SD OCT, may be correlated with subclinical visual disability, and may therefore serve as a new efficient objective clinical metric for diagnosing early atrophic AMD as well as serving as a new adjunct measure of progression. 

## 2. Methods

Subjects were included from two data sets: a subset of the Zeaxanthin and Vision Function (ZVF) study data set and an additional de-identified clinical OCT data acquired within the Eye Clinic, DVA Medical Center, North Chicago, IL, during routine clinical care. ZVF is a prospective 12 month *n* = 119 eye, randomized, placebo controlled (RCT) trial evaluating one degree macular pigment optical density (MPOD) and distribution (MP), visual acuity, CSF, glare recovery, shape discrimination, large field B/Y color vision threshold, and lipofuscin pattern distributions following supplementation with the carotenoids: zeaxanthin at 8 mg, lutein at 9 mg or a combination of both. ZVF was approved by the R&D and Human Subjects Committees of the Department of Veterans’ Affairs Medical Center, Hines, IL under the auspices of the FDA (www.ClinicalTrials.Gov IND#78.973; Richer *et al.* [10]). A total of 56 eyes of twenty nine subjects (27 males and 2 females) had completed ZVF with available post-research study SD OCT scans. In the case of the second larger observational SD OCT data set, de-identified clinical data on a random subset of AMD clinic patients was merged with the ZVF 12 month visual function data subset. Thus SD OCT scans of these 29 Clinic subjects, 50 to 88 years (73 ± 10.5 years) of age with mild atrophic AMD cases (but without vision function data) were merged with the ZVF subjects to create a second larger data set consisting of 98 eyes of fifty four subjects (49 males and 5 females) with an age range of 50 to 91 years (74 ± 10.1 years). Only patients with atrophic AMD and typical SD OCT retinal signs were included. For example, RPE/Photoreceptor or photoreceptor integrity line (PIL) disruption, soft drusen, drusenoid RPE detachments, outer nuclear layer reflective (inflammatory) bodies, *etc.* but without evidence of sensory retinal detachment or sub-retinal neovascularization [6]. Subjects with the entity “Age-Related Choroidal Atrophy” manifesting extreme choroidal thinning of less than 125 microns were excluded [11]. Potential retinal thickness and visual function confounders, such as subjects with glaucoma, significant diabetic retinopathy (*i.e*., macular edema), pre-retinal gliosis and vitreal traction syndrome were excluded. In the ZVF group, four patients had both eyes and three patients had only one eye excluded whereas in the eye clinic AMD patient group, seven patients had both eyes excluded. Subjects were limited to ±6.00 DS refractive error. Informed consent was obtained from ZVF subjects and the Declaration of Helsinki protocols were maintained.

**Figure 2:**
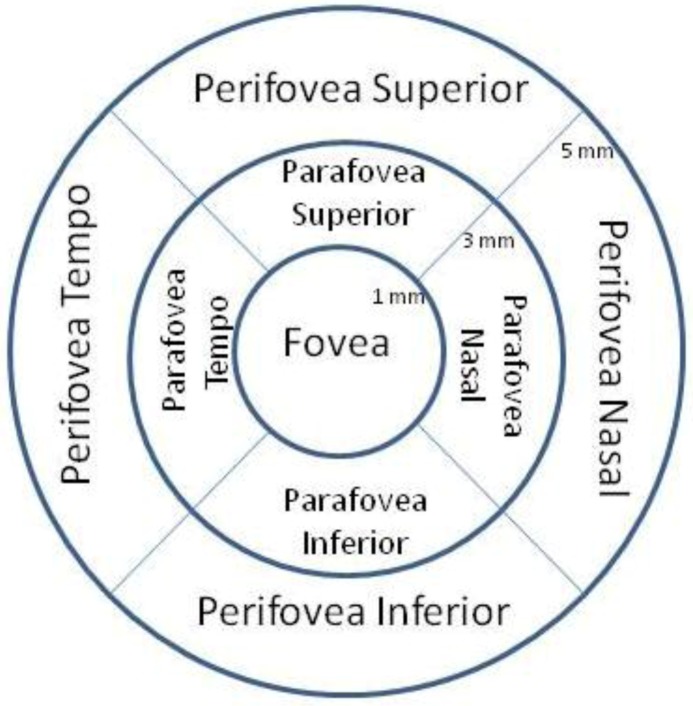
Full thickness SD OCT MM5 EDTRS significance map schematic. Nine zone ETDRS (Early Treatment of Diabetic Retinopathy) grid provided by the RTVue SDOCT measures an area 5mm × 5mm on the retina (MM5 retinal thickness scan pattern). This grid is centered on the fovea and shows the relative location of each of four extra-foveal quadrants defined as the sum of the parafovea + perifovea regions (figure courtesy Optovue Inc., Fremont, CA).

The RTVue provides a detailed sampling of over 19,000 thickness points plotted in a 5 mm × 5 mm area of the central macula. The Full Thickness MM5 Significance Map presentation reveals the significance of the full retinal thickness deviation from normal, of the scanned retina (Figure 1a). The total area of significant loss (blue areas in the Significance Map) was calculated and divided by the total area of the Map to calculate the percent extrafoveal thinning (% EFT), and used for functional correlation. The MM5 provides nine retinal thickness measurements based on the ETDRS, is centered on the fovea with a diameter of 1 mm, four *parafoveal quadrants* surrounding the central fovea extending form 1 mm out to 3 mm, and four perifoveal *quadrants* extending from 3 mm to 5 mm. Extra foveal paramaters refer to the sum of *parafoveal *+ *perifoveal *area. Figure 2 depicts these nine regions**.** An age-matched subset of the RTVue normative database was used for comparison. Normative data from 309 patients (594 retinas) age 50–82 were available. The subfoveal choroidal thickness was measured using the En Face viewing option of the 3D macular presentation. This view presents the sum of all c-scan planes of the same 5 mm macular radius in a top-down fundus view. The choroidal layer was determined to be the distance between Bruch’s membrane to a depth at which choroidal vessels can no longer be seen. Two measurements were made for each eye, with an acceptable intra-observer (author JC) coefficient of variation of +0.90. The average choroidal thickness of these two measurements is reported. 

Visual psychophysical data, lens opacification and NEI VFQ25 vision questionnaire and retinal grading were available for ZVF patients only. Best refracted distance visual acuity measured with randomly presented ETDRS letters on an M&S Technologies (Chicago, IL) SmartSystem II LCD monitor viewed at 10 feet (SR). The CSF test was performed using best refracted maximum visual acuity and the Functional Vision Anayzer (Stereo Optical, Inc Chicago, IL) as previously described [12,13]. Blue/Yellow Color Vision increment thresholds were ascertained with the ChromaTest^®^ (Bromley, UK) with best corrective lenses according to protocol [10,14,15]. Integrated 6 degree diameter Macular Pigment (MP) area under the curve (AUC) was determined objectively with an ARIS 110 camera (Visual Pathways, Prescott, AZ) by the method of specular reflectance [16]. 

Lens opacification, a confounder of CSF, was qualitatively assessed using a seven increment scaled LOCSIII transparency with multivariate analysis as previously described [9,12,13]. Fifty degree fundus images were taken with a Kowa VX-10 (Tokyo, Japan). Retinal grading of AMD disease state was completed in a double-masked randomized fashion by a retinal specialist (ML) utilizing the AREDS report #18 simplified (0–4) scale for presence of hypo/hyper RPE pigmentation and soft drusen [17]. Overall visual function was assessed with the NEI VFQ25 vision function questionnaire [10].

Statistics: As with ZVF [10], average-eye visual function data from a single subject, and significance, is typically shown for simplicity, in cases in which the analysis did not differ appreciably from evaluating right eyes and left eyes separately. Thus the last three figures in the RESULTS section present data for both eyes. Statistical analysis utilized two sided, student *T*-tests for paired comparison data and *P* < 0.05 was considered significant. 

## 3. Results

**Figure 3:**
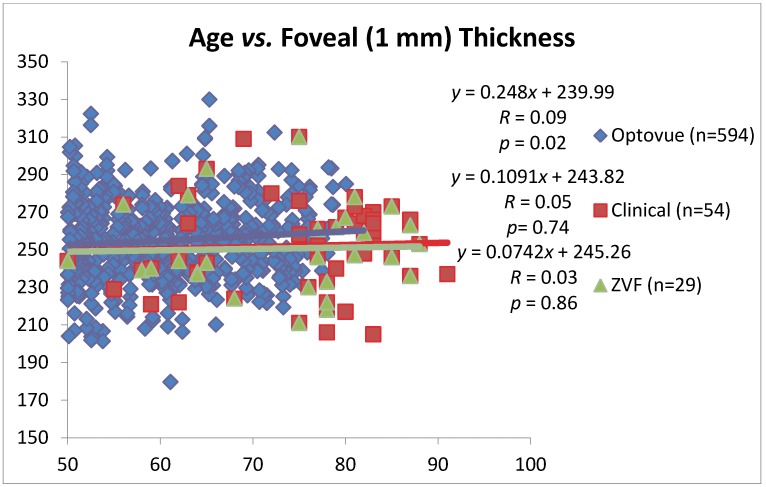
Foveal thickness and aging. RTVue SD OCT normative retinal thickness MM5 data from (*n* = 597) international retinas of an all ethnicities and gender data base between the ages of 50 and 82. The normal fovea thickens approximately 2.5 μm/decade. (Courtesy Optovue, Fremont, CA) Also shown is retinal thickness *vs.* age for post study ZVF AMD subjects (*n* = 29 retinas) and a combined AMD clinical patient population inclusive of theseresearch (*n* = 54 retinas) with a greater number of AMD subjects in the 82–91 age group.

The right, left and average eye AMD simplified AREDS grade retinopathy for the ZVF fundus image data set was 0.71 (SD 0.6)/2.0 max score, 0.71 (SD 0.5) 2.0 max score, and 1.43 (SD 0.92)/4.0 max bilateral retinal score signifying mild AMD. Average eye ETDRS visual acuity was 20/20-1 (SD 1.2 lines). Figure 3 is a scatter plot of age *vs.* average foveal thickness from *n* = 594 “Normal” retinas from patients ages 50–82 of various ethnicities. This Optovue normative data suggests a slight foveal thickening with age (*r* = 0.09, *P* = 0.02). AMD patients from the ZVF study (*n* = 29 AMD retinas) and the larger merged clinical/research AMD data set (*n* = 54 AMD retinas), ages 50+ reveal: (1) slightly thinner foveal thickness compared to these “Normals” and (2) a thickness that does not change with age. Regardless, “Normal” and AMD foveal thickness appears to be highly guarded, conserved, and little changed with age. 

**Figure 4:**
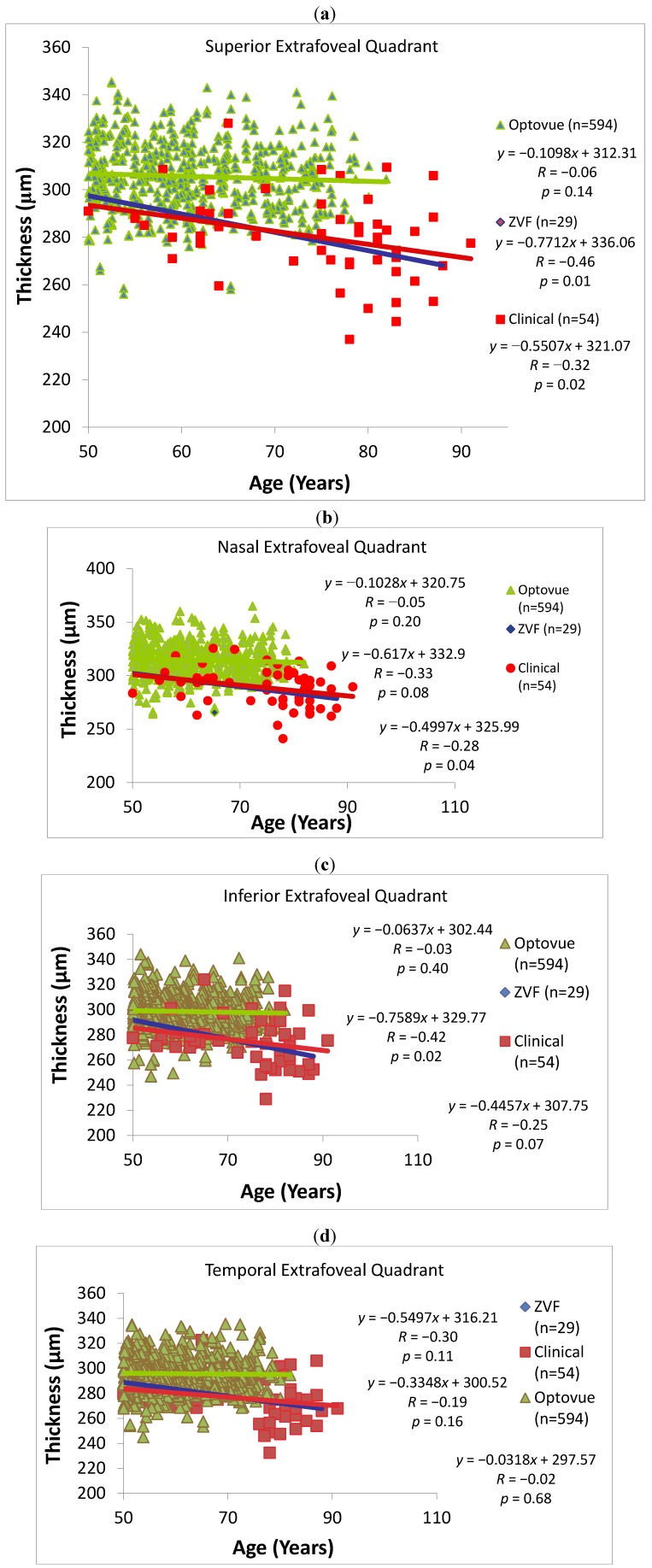
Extra-foveal quadrant thickness *vs.* age. (**a**) Superior quadrant; (**b**) Nasal quadrant; (**c**) Inferior quadrant; (**d**) Temporal quadrant. Plots reflect the RTVue SD OCT age matched normative data against the two AMD retina data sets: ZVF and the larger clinical AMD patient data set that includes ZVF study patients. Both AMD data sets display accelerated thinning with age, especially for the superior quadrant (*P* < 0.02). See Table 1.

In contradistinction to this slight foveal thickening, Figure 4a–d and Table 1 show parafoveal + perifoveal quadrants slightly *thin *with age in normals, although not significantly: (Superior Quadrant: −1.1 μm/decade; Nasal Quadrant: −1.0 μm/decade; Inferior Quadrant −0.6 μm/decade and Temporal Quadrant −0.3 μm/decade). In both the ZVF AMD study and the larger merged AMD clinical data set, overall macular thickness excluding the fovea show accelerated thinning at a rate of −6.7 μm/decade (*P* = 0.03) and −4.6 μm/decade (*P* = 0.04) respectively. Specifically, AMD patients thin by −5.5 μm/decade (*P* < 0.02) superiorly, −5.0 μm (*P* < 0.04) nasally, −4.5 μm (*P* < 0.07 for trend) inferiorly, and −3.3 μm (NS) temporally using data from 54 AMD retinas. Table 1 summarizes this AMD retinal thinning effect. This difference is outside the 5 μm coefficient of variation of the instrument. More specifically, Table 2 depicts accelerated thinning of macular retinal thickness (μm/decade) in the overall 5 mm retinal diameter ETDRS diameter grid *excluding the fovea *and extrafoveal quadrants (but not the fovea) in AMD retinas, compared with the age matched “Optovue normative” population data. A statistically significant AMD retinal thinning effect is particularly evident in the vulnerable superior retinal ETDRS quadrant compared with the temporal quadrant (ns).

**Table 1:** The mean (SD) retinal thickness (µm) of patients with AMD is thinner than those from the OptoVue SD OCT “normative” population data base. This difference is outside the 5 µm axial published instrument coefficient of variation.

	Superior	Nasal	Inferior	Temporal
AMD Clinic (*n* = 54 eyes)	280 ± 17.5 µm	288.7 ± 18	274.5 ± 18	275.5 ± 17.4
AMD ZVF (*n* = 29 eyes)	279.6 ± 17.7	287.7 ± 19.5	274.2 ± 19.1	276 ± 19.3
Normative (*n* = 594 eyes)	305.6 ± 14.6	314.4 ± 15.6	298.5 ± 14.8	295.6 ± 14.7

**Table 2:** Accelerated thinning of macular retinal thickness (μm/decade) in the overall 5mm retinal diameter ETDRS diameter grid *excluding the fovea and *extrafoveal quadrants (but not the fovea) in ZVF Study and clinical AMD retinas, compared with the age matched “Optovue normative” population data. A statistically significant thinning effect in AMD is particularly evident in the vulnerable superior retinal parafoveal + perifoveal ETDRS quadrant [10], see text.

Region	ZVF AMD Studay (*n* = 29) μm/decade	Clinical AMD Patients (*n* = 54) μm/decade	Optovue “Normal” Data ^1^ (*n* = 594) μm/decade
Overall (Excluding Fovea)	−6.7 * (*P* = 0.03)	−4.6 * (*P* = 0.04)	−0.8 (*P* = 0.30)
Superior	−7.7 * (*P* < 0.01)	−5.5 * (*P* = 0.02)	−1.1 (*P* = 0.14)
Nasal	−6.2 (*P* = 0.08)	−5.0 * (*P* = 0.04)	−1.0 (*P* = 0.20)
Inferior	−7.6 * (*P* = 0.02)	−4.5 (*P* = 0.07)	−0.6 (*P* = 0.40)
Temporal	−5.5 (*P* = 0.11)	−3.3 (*P* = 0.19)	−0.3 (*P* = 0.68)
Fovea	+0.7 (*P* = 0.86)	+1.1 (*P* = 0.74)	+2.5 (*P* = 0.09)

* Indicates significant data; ^1^ Optovue, Inc., Fremont, CA, normative database.

**Figure 5:**
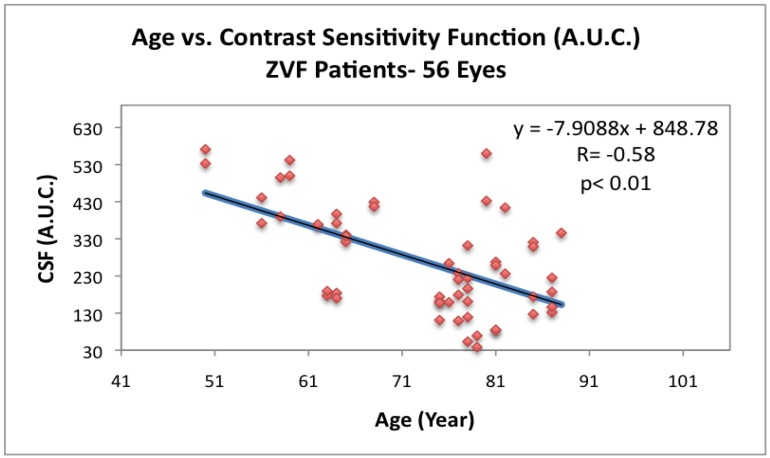
Contrast sensitivity *vs.* age for ZVF AMD subjects. Loss of CSF with age in *n* = 56 AMD eyes from the ZVF study. Integrated area under the curve of the contrast sensitivity function (CSF) at four spatial frequencies: 3 cc, 6 cc, 12 cc and 18 cc/degree [10]. (Functional Vision Analyzer^®^, Stereo Optical, Inc., Chicago,IL).

**Figure 6:**
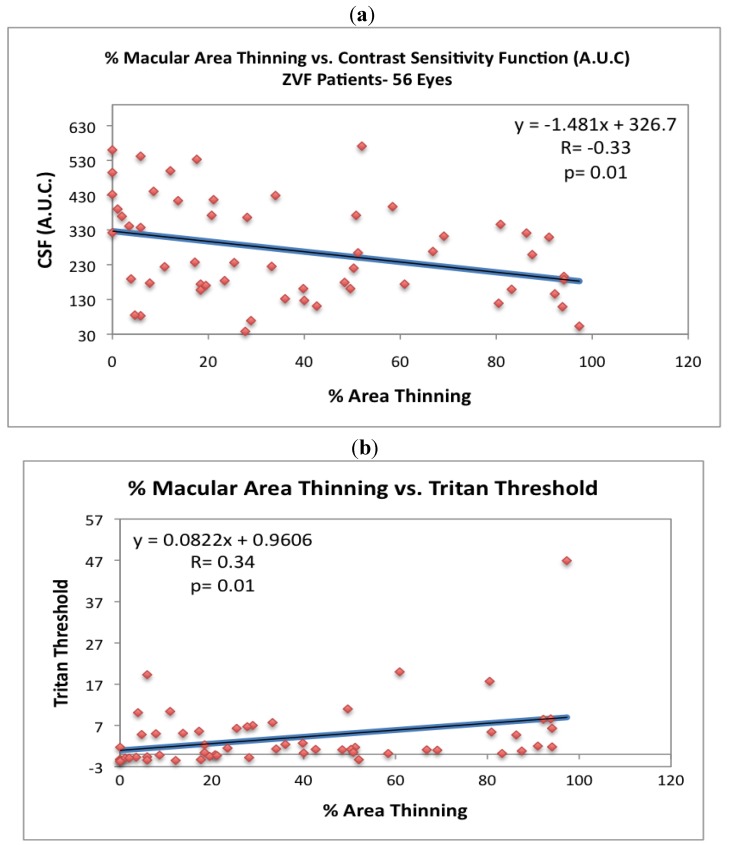
The visual consequences of extra-foveal thinning. (**a**) Contrast sensitivity (area under the curve) is inversely related to % retinal thinning (*r* = −0.33 *P* = 0.01, 2 Tailed paired *T* test). (**b**) 6.5 degree increment blue-threshold is directly related to % retinal thinning (*r* = +0.34, *P* = 0.01, 2 Tailed paired *T* test). Blue/Yellow Color Vision increment thresholds were ascertained with the ChromaTest^®^ (Bromley, UK) with best corrective lenses according to protocol [10,14,15].

Figure 5 depicts loss of CSF with age for the ZVF AMD subjects. Parenthetically, the NEI Vision Function Questionnaire (VFQ 25) total all category summed score (data not shown) was positively correlated with this loss of contrast sensitivity (*r* = +0.27, *P* = 0.04) and inversely correlated with % total retinal thinning but not significantly (*r* = −0.20, *P* = 0.13). As shown in Figure 6a, CSF is inversely related to % macula thinning (*r* = −0.33, *P* = 0.01, 2 Tailed T Paired Comparison) , while Figure 6b depicts the 6.5 degree ChromaTest^®^ B/Y threshold to be heightened and directly related to % macula thinning (*r* = +0.34, *P* = 0.01, 2 Tailed T Paired Comparison). 

**Figure 7:**
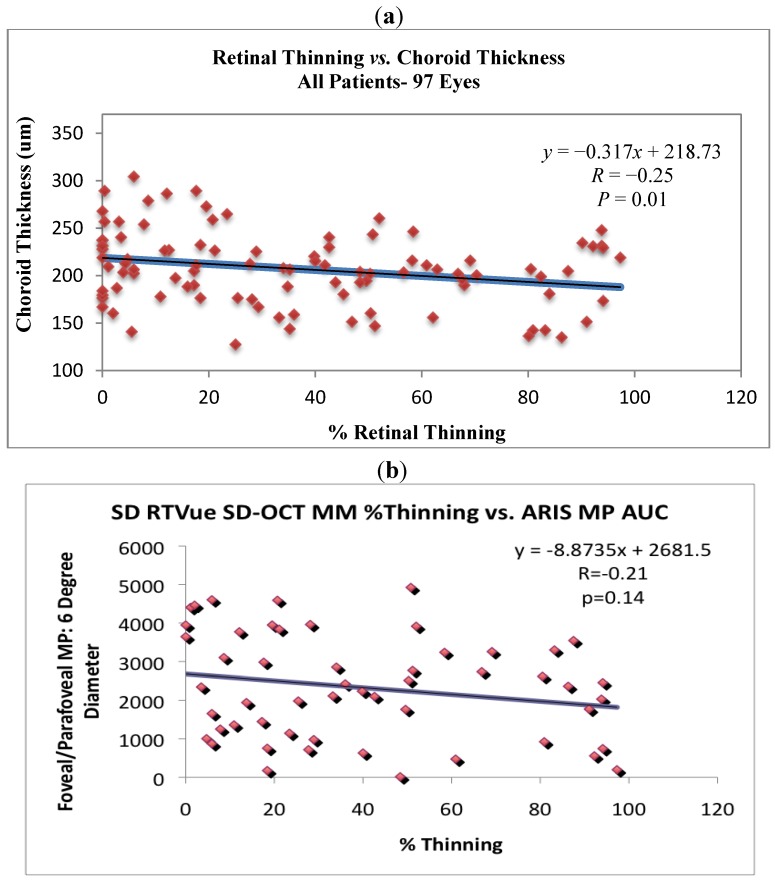
Retinal and choroidal thinning are associated. (**a**) SD OCT measure of choroidal thickness *vs.* % retinal thinning. The coefficient of variation with the manual caliper method, same observer, was *r* = 0.90. Note the significant negative correlation between % retinal thinning and choroidal thickness (*r* = −0.25, *P* = 0.01) for all subjects. (**b**) Three dimensional macula pigment optical density (integrated volume) specular reflectance measurement (ARIS 110 camera, Visual Pathways, Prescott, AZ) *vs.* retinal thinning, reveals % extrafoveal thinning to be associated with declining volumetric macular pigment optic density (MPOD), but not significantly.

Retinal thickness (excluding the fovea) correlated with choroidal thickness, but not significantly (*R* = +0.15, *P* = 0.13). However, Figure 7a shows a significant negative correlation between % thinning and choroidal thickness (*r* = −0.25, *P* = 0.01) for all subjects while Figure 7b suggests that parafoveal/perifoveal thinning is associated with declining overall volumetric macular pigment optical density (MPOD), but not significantly (*r* = −0.21, *P* = 0.14). Precise three dimensional MPOD data was available only for ZVF subjects. 

## 4. Discussion

Foveal thickness appears well protected with age. Even our early AMD patients had no change in central foveal thickness with age. One might expect one or more of the following factors to be associated with age-related retinal thinning: (1) retinal atrophy; (2) choroidal atrophy; or (3) lower foveal macular pigment. We believe the third explanation is germane to this report, as all of the subjects, both post ZVF Study subjects and clinical patients, despite their advancing age had supplemented with approximately 10 mg or more of the oral retinal carotenoids lutein and/or zeaxanthin.

In a manner analogous to neural retinal rim thinning or more recently the concept of pre-glaucomatous ganglionic layer (GCC) cell loss in glaucoma, the atrophic extrafoveal macula appears to thin in AMD at a greater rate than predicted by simple aging. Confounding high myopia was excluded from our database. The order effect with superior most vulnerable and temporal least vulnerable was shown in two data sets—a research data set and a merged research + clinical data set. This pathological patterned loss of tissue was first eloquently revealed by Sarks, Sarks and Killingsworth decades ago using serial fluorescein angiography, white light, and electron microscopy [18]. Our results agree with that historic study.

Our observation that atrophic AMD typically begins in the superior parafovea (at approximately 7 degrees eccentricity) has been confirmed by two other research groups and at the ARVO 2010 meeting (Ft Lauderdale, FL). Significantly, at the Oregon National Primate Research Center, Rhesus monkeys deprived of carotenoids from birth were found to develop atrophic AMD in an extra-foveal superior location [19]. A second group from the Australian National University, using a novel pupillary reaction test, mapped human AMD functional deficits in sector pupillary latency to the superior parafovea [20]. Furthermore, it has been suggested, that the superior half of the retina is vulnerable due to the damaging effects of reflective UV ambient ground reflecting radiation experienced by humans as primates typically walk with a slight head-down gaze (Personal Communication [21]). Ascertaining the health of the superior parafoveal rod rich retina is the basis of the new Maculogix (Atlanta, GA) AMD detection device. 

A limitation of this study is the dearth of “normative” patients in the age 82–91 range (Figure 3) combined with the high likelihood of unrecognized subclinical AMD in the “normative” data base especially in old old “normals”. However, this lack of patients in the oldest age group combined with the fact that our AMD subjects all had visible retinal pathology creates a counterbalancing bias. This report further supports the conclusion of both the Lutein Antioxidant Supplementation Trial (LAST) and recently published ZVF Study, that subtle signs of photoreceptor—retinal pigment epithelium disturbance characteristic of AMD, such as CSF and increment B/Y color vision thresholds, are impaired long before the appearance of obvious ophthalmoscopic or non-spectral photographic AMD pathological signs appear (*i.e*., soft drusen or RPE rarefaction). That is, functional visual decline *precedes* photoreceptor—retinal pigment epithelium atrophy [10,12]. The AMD patients in this report had early disease with an AREDS Report #18 five year risk estimate of only 8% for future catastrophic monocular vision loss, near perfect 20/20 ETDRS visual acuity, and no fovea retinal thinning. 

In our combined clinical and research experience, patients with even extreme extra-foveal retinal thinning might go undetected in current clinical practice where CSF or even simple, quick near point low contrast Colenbrander visual acuity screening is seldom utilized except perhaps in refractive surgical and contact lens practices [22]. It makes sense that large field (6.5 degree) blue sensitivity suffers earliest in AMD because blue cones are fragile and few in number [14]. By the time the patient is in danger of an acute degenerative change, a flashed 6.5 degree diameter blue/yellow stimulus optotype becomes indistinguishable no matter how intense the color. Once again, subtle color vision disturbances in early AMD are rarely evaluated except in the research laboratory. The decline in visual function in early AMD is far from academic. CSF is related to driving safety and hip fracture risk as well as overall quality of life with mortal implications [23,24,25]. While a 10-letter (two-line) decrease in best corrected VA is significantly associated with all self-reported measures of visual disability, a mere 2-step decrease in contrast sensitivity is significant [25]. 

There is increasing recognition that the optical and antioxidant properties of the xanthophyll pigments lutein, zeaxanthin, and lutein’s metabolite mesozeaxanthin, play an important role in maintaining the health and function of the human macula [16]. Foveal MPOD has a significant positive, measurement technique independent relationship of approximately *r* = +0.30 with central retinal thickness [26]. Significantly, in that study there was no demonstrable relationship between MPOD at an eccentricity of one or two degrees and central retinal thickness. However in the present AMD study, Figure 7b shows a non-significant trend for extra-foveal thinning to be associated with declining volumetric macular pigment. We also found no significant change in central foveal thickness in agreement with the excellent preservation of visual acuity in both our research and clinical population. Interestingly, several groups have established that MPOD tends to decrease with age [16]. This is conjectured to contribute to the accelerated retinal thinning we observed ostensibly through either direct deposition (*i.e*., de-pigmentation) or by secondary indirect declining antioxidant tissue protection of the retina and the choriocapillaris/choroidal vascular bed [27]. We also found a significant association between choroidal thinning and retinal thinning. Spaide describes a decrease of 16 μm/decade choroidal thickness in high myopes [11]. Our ZVF AMD patients showed an even greater 22 μm/decade decrease in choroidal thickness with each decade (*r* = −0.57; *P* < 0.01).

## 5. Conclusion

New technologies and techniques are required to evaluate the effect of nutrient intervention on retinal health in early and moderate AMD. In this report, we present a new objective SD OCT based atrophic AMD metric called “% extra-foveal retinal thinning” or % EFRT. We have shown that % EFRT is associated with loss of contrast sensitivity and extra-foveal blue cones in early AMD. Macular thickness ETDRS SD OCT thickness plots provide eye practitioners with a new clinically useful and objective surrogate measure of early functional visual loss of aging retinas. Indeed, the data and simulations presented in this report explain why practitioners often underestimate the functional impact of a “20/20 AMD diagnosis”. Researchers can employ CSF/Low Contrast Screening, SD OCT and MP distribution instrumentation together, to gauge overall and distributional changes in retinal thickness and formally evaluate how prescriptive carotenoid repletion (*i.e*., lutein/mesozeaxanthin and zeaxanthin along with synergistic *n*-3 fats) impacts % EFRT and visual function. This new metric can be applied to the study of aging, genetic susceptibility and age related choroidal degeneration. The authors believe % EFRT to be superior to the following three currently employed tests: the 150 year old Snellen chart, the 117 year old Amsler Grid and conventional non-spectral fundus cameras. Significantly, % EFRT has potential to meaningfully and efficiently evaluate both nutritional and pharmacologic atrophic AMD intervention(s) in clinical practice as well as retinal research. 
